# A [4Fe-4S] cluster resides at the active center of phosphomevalonate dehydratase, a key enzyme in the archaeal modified mevalonate pathway

**DOI:** 10.3389/fmicb.2023.1150353

**Published:** 2023-03-13

**Authors:** Mutsumi Komeyama, Kohsuke Kanno, Hiroyuki Mino, Yoko Yasuno, Tetsuro Shinada, Tomokazu Ito, Hisashi Hemmi

**Affiliations:** ^1^Graduate School of Bioagricultural Sciences, Nagoya University, Nagoya, Aichi, Japan; ^2^Graduate School of Science, Nagoya University, Nagoya, Aichi, Japan; ^3^Graduate School of Science, Osaka Metropolitan University, Sugimoto, Osaka, Japan

**Keywords:** iron–sulfur cluster, archaea, mevalonate pathway, electron paramagnetic resonance, isoprenoid

## Abstract

The recent discovery of the archaeal modified mevalonate pathway revealed that the fundamental units for isoprenoid biosynthesis (isopentenyl diphosphate and dimethylallyl diphosphate) are biosynthesized *via* a specific intermediate, *trans*-anhydromevalonate phosphate. In this biosynthetic pathway, which is unique to archaea, the formation of *trans*-anhydromevalonate phosphate from (*R*)-mevalonate 5-phosphate is catalyzed by a key enzyme, phosphomevalonate dehydratase. This archaea-specific enzyme belongs to the aconitase X family within the aconitase superfamily, along with bacterial homologs involved in hydroxyproline metabolism. Although an iron–sulfur cluster is thought to exist in phosphomevalonate dehydratase and is believed to be responsible for the catalytic mechanism of the enzyme, the structure and role of this cluster have not been well characterized. Here, we reconstructed the iron–sulfur cluster of phosphomevalonate dehydratase from the hyperthermophilic archaeon *Aeropyrum pernix* to perform biochemical characterization and kinetic analysis of the enzyme. Electron paramagnetic resonance, iron quantification, and mutagenic studies of the enzyme demonstrated that three conserved cysteine residues coordinate a [4Fe-4S] cluster—as is typical in aconitase superfamily hydratases/dehydratases, in contrast to bacterial aconitase X-family enzymes, which have been reported to harbor a [2Fe-2S] cluster.

## 1. Introduction

The archaeal mevalonate (MVA) pathway is a modified version of the eukaryotic MVA pathway (Hayakawa et al., 2018; Supplementary Figure 1). In this recently discovered pathway, (*R*)-mevalonate 5-phosphate (MVA5P) is converted into *trans*-anhydromevalonate phosphate (tAHMP), a specific intermediate of the pathway, by phosphomevalonate dehydratase (PMDh). tAHMP is then decarboxylated and phosphorylated to yield isopentenyl diphosphate, a common precursor for the biosynthesis of isoprenoids, such as archaeal membrane lipids. This pathway is utilized by archaea, excluding some exceptions such as halophilic archaea of the class Halobacteria (Dellas et al., 2013; VanNice et al., 2014) and thermoacidophilic archaea of the orders Sulfolobales (Nishimura et al., 2013) and Thermoplasmatales (Azami et al., 2014; Vinokur et al., 2014, 2015, 2016; Rossoni et al., 2015; Aoki et al., 2022); therefore, it is considered the evolutionary prototype of the eukaryotic, haloarchaea, and *Thermoplasma*-type MVA pathways (Hayakawa et al., 2018; Aoki et al., 2022). The archaeal MVA pathway is also characterized by its lower ATP consumption compared to the other MVA pathways (Hayakawa et al., 2018). The energetically lower cost of the biosynthesis of isoprenoid precursors would be a critical factor in the metabolic engineering of microbial production of isoprenoids.

**Figure 1:**
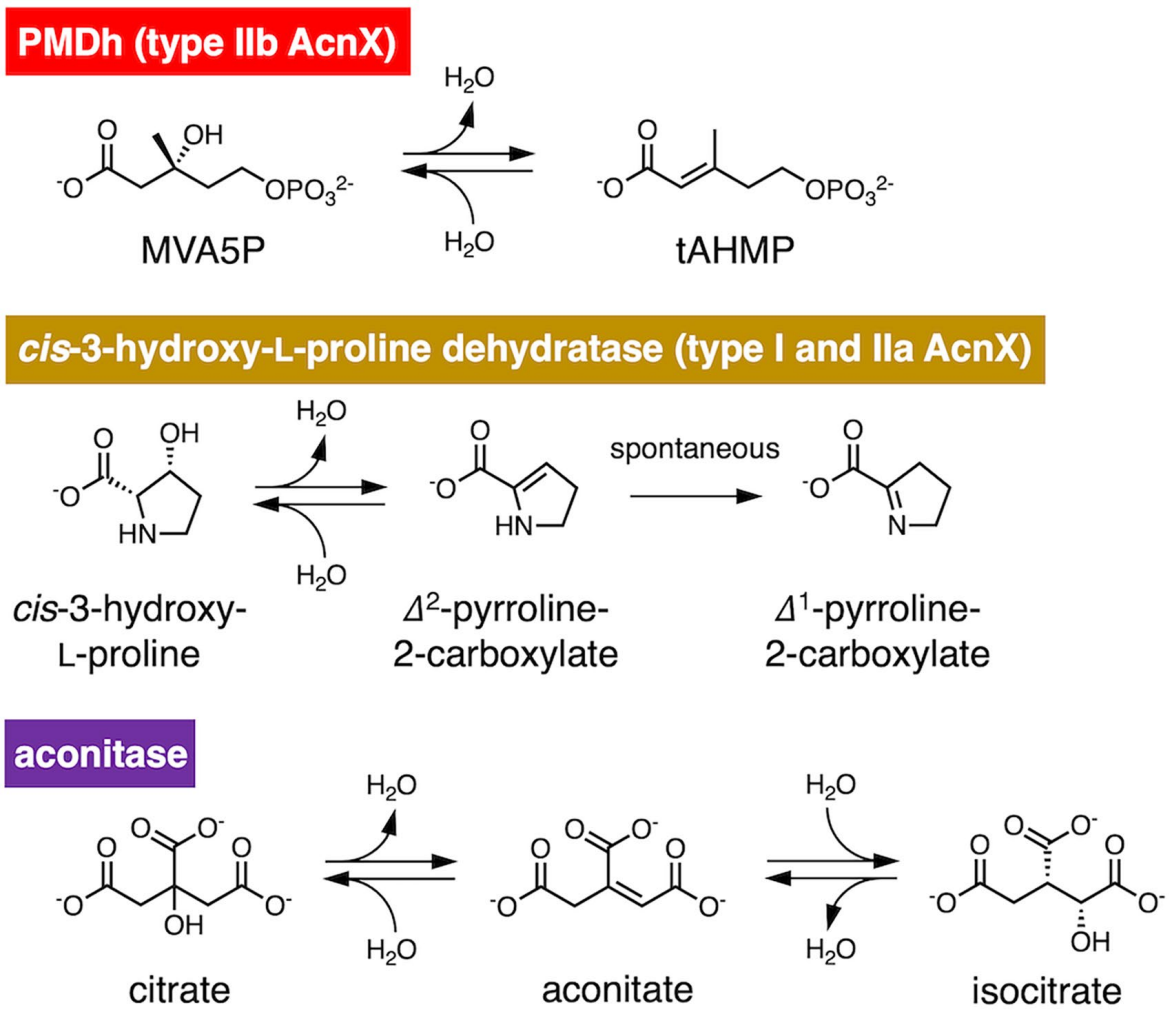
Reversible dehydration/hydration reaction catalyzed by phosphomevalonate dehydratase (PMDh), its bacterial homolog in the aconitase X (AcnX) family, and aconitase. PMDh assay in this study was performed *via* the hydration reaction from *trans*-anhydromevalonate phosphate (tAHMP) to (*R*)-mevalonate 5-phosphate (MVA5P).

PMDh, a key enzyme in the archaeal MVA pathway, was first isolated from the hyperthermophilic archaeon *Aeropyrum pernix* (Hayakawa et al., 2018; Figure 1). *A. pernix* PMDh (ApPMDh) catalyzes the conversion of MVA5P into tAHMP, with equilibrium biased to MVA5P. Quantitative analysis of ApPMDh reactions showed that 87.7 ± 4.6% of tAHMP was converted into MVA5P in an equilibrium achieved by reverse reaction (Yasuno et al., 2021). Recombinant *A. pernix* PMDh (ApPMDh) exhibited a pale brown color immediately after purification from *E. coli* cells, but the color typically diminished after a few hours of air exposure. The decolorized recombinant protein lost most of its catalytic activity. The activity of PMDh from the methanogenic archaeon *Methanosarcina mazei* has also been confirmed by the successful reconstitution of a part of the archaeal MVA pathway in *Escherichia coli* cells, which was demonstrated visually by an increase in carotenoid production; however, an *in vitro* assay of the enzyme has not been performed (Yoshida et al., 2020).

**Figure 2:**
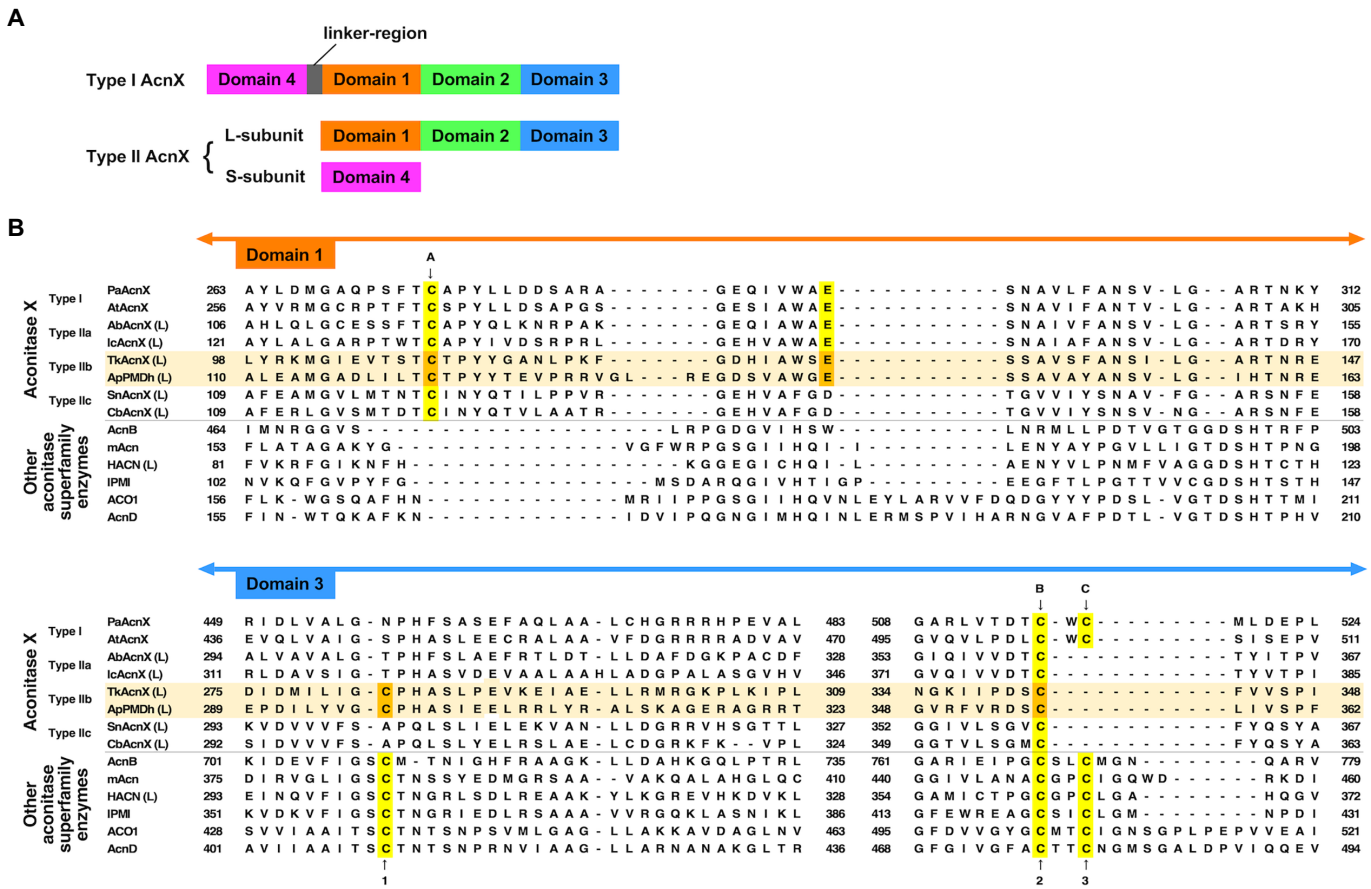
Characteristics of the primary structures of type IIb aconitase X (AcnX) enzymes and their homologs. **(A)** Domain structures of AcnX family enzymes. **(B)** Multiple sequence alignment of AcnX family and other aconitase superfamily enzymes. Sequence alignment was performed online using MAFFT with default parameters (https://mafft.cbrc.jp/alignment/server/; Katoh et al., 2019). The sequences of Type IIb AcnX are highlighted in pale orange. The conserved cysteine residues corresponding to those involved in iron–sulfur cluster binding in type I AcnX (cites A–C) and in aconitase superfamily enzymes (cites 1–3), as well as the conserved glutamate residues corresponding to that required for the activity of type I AcnX, are highlighted in yellow (or orange in the case of type IIb AcnX). The orange-colored residues in *Aeropyrum pernix* PMDh (ApPMDh), i.e., Cys122, Glu145, Cys297, and Cys356, were mutagenized in this study. PaAcnX, *cis*-3-hydroxy-L-proline dehydratase from *P. aeruginosa*; AtAcnX, *cis*-3-hydroxy-L-proline dehydratase from *Agrobacterium tumefaciens*; AbAcnX, *cis*-3-hydroxy-L-proline dehydratase from *Azospirillum brasiliense*; IcAcnX, putative *cis*-3-hydroxy-L-proline dehydratase from *Intrasporangium calvum*; TkAcnX, putative PMDh from *Thermococcus kodakarensis*; SnAcnX, AcnX family protein from *Starkeya novella* with unknown function; CbAcnX, AcnX family protein from *Cupriavidus basilensis* with unknown function; AcnB, *Escherichia coli* aconitase B; mAcn, mitochondrial aconitase from bovine; ACO1, cytosolic aconitase from human; AcnD, 2-methylcitrate dehydratase from *Shewanella oneidensis*; HACN (L), homoaconitase large subunit from *Methanocaldococcus jannaschii*; IPMI, isopropylmalate isomerase from *Saccharomyces cerevisiae*.

PMDh belongs to the aconitase X (AcnX) enzyme family, which is part of the aconitase superfamily (Makarova and Koonin, 2003; Figure 2). Although the aconitase superfamily includes well-studied enzymes such as aconitase (see also Figure 1), homoaconitase (*cis*-homoaconitate dehydratase), and isopropylmalate dehydratase (Gruer et al., 1997), the AcnX family is a group of homologous enzymes whose functions have only recently been elucidated (Watanabe et al., 2016, 2017; Hayakawa et al., 2018). The AcnX family was discovered in a comparative genomic study (Makarova and Koonin, 2003), and comprises several subfamilies: type I, type IIa, type IIb, and type IIc-f (Watanabe et al., 2016, 2017, 2021). The type I AcnX subfamily contains *cis*-3-hydroxy-L-proline dehydratases (Figure 1) from bacteria, such as *Pseudomonas aeruginosa* and *Agrobacterium tumefaciens*, and fungi, such as *Trichoderma reesei* (Watanabe et al., 2016). The type IIa subfamily also contains bacterial *cis*-3-hydroxy-L-proline dehydratases (Watanabe et al., 2017), whereas the type IIb subfamily contains archaeal PMDhs (Hayakawa et al., 2018; Yoshida et al., 2020). The functions of bacterial enzymes belonging to other type II subfamilies (type IIc–IIf) remain unknown. Type I AcnX is encoded by a single gene, and its 4–1–2-3 domain organization resembles that of bacterial aconitase B (Figure 2A). Type II AcnX enzymes are composed of large (corresponding to domains 1–2-3 of aconitase) and small (corresponding to domain 4) subunits, like archaeal and bacterial homoaconitases and isopropylmalate dehydratases, and the subunits are usually encoded in tandemly aligned or neighboring genes. One [4Fe-4S] cluster is known to be contained in tightly packed domains 1–2-3 of most aconitase superfamily enzymes (Lauble et al., 1992; Gruer et al., 1997; Williams et al., 2002; Volz, 2008). The [4Fe-4S] cluster of most aconitase superfamily enzymes is coordinated with three conserved cysteine residues in domain 3 (at sites 1–3 in Figure 2B). Each cysteine residue binds to the iron of the cluster *via* its thiol group, and a vacant iron acts as a catalyst in dehydration/hydration reactions (Lauble et al., 1992).

However, a recent report by Watanabe et al. (2021) contradicts this common assumption. The authors determined the crystal structures of type I AcnX from *A. tumefaciens* (pdb#:7CNP and 7CNQ), which has *cis*-3-hydroxy-L-proline dehydratase activity, and those of type IIb AcnX from a hyperthermophilic archaeon *Thermococcus kodakarensis* (7CNR and 7CNS), although the enzymatic activity of the PMDh homolog was not confirmed. Surprisingly, the structures of *A. tumefaciens* type I AcnX had a [2Fe-2S] iron–sulfur cluster. The existence of the [2Fe-2S] cluster was also supported by electron paramagnetic resonance (EPR) analysis of the enzyme and its homolog from *Pseudomonas* sp. (Watanabe et al., 2021) In the substrate-free structure of *A. tumefaciens* type I AcnX (7CNP), the cluster is coordinated by three cysteine residues, Cys129, Cys508, and Cys510 (at sites A–C in Figure 2B), and a water molecule, which is replaced by the substrate *cis*-3-hydroxy-L-proline in the substrate-complex structure (7CNQ). All cysteine residues are conserved in type I AcnXs, but their positions are not completely identical to those of the conserved cysteines in the aconitase superfamily enzymes, as shown in Figure 2B. The cysteine residue at site A (Cys129 in *A. tumefaciens* AcnX) is not conserved in most aconitase superfamily enzymes, whereas those at site B (Cys508) and site C (Cys510) correspond well with the conserved cysteine residues at sites 2 and 3, respectively. In contrast, the crystal structures of the putative PMDh from *T. kodakarensis* (TkPMDh) contain a [3Fe-4S] cluster, and EPR analysis of the dithionite-reduced putative PMDh provided no evidence of the presence of a [4Fe-4S] cluster (Watanabe et al., 2021). However, it is suspected that the putative PMDh binding to the [3Fe-4S] cluster is in an inactive state, as no enzyme activity was shown. The sequences of type I and type IIb AcnXs also differed considerably. The cysteine residue at site C does not exist in PMDh homologs, whereas those at sites A and B are well conserved (Figure 2B). Instead, PMDh homologs have another conserved cysteine residue at the position corresponding to that in aconitases at site 1. This confusing situation—in which different ion-sulfur clusters are formed in homologous enzymes—motivated us to identify the structure of the iron–sulfur cluster bound to active PMDh from *A. pernix*.

In the present study, we performed iron–sulfur cluster reconstruction to activate purified ApPMDh. The basic properties of ApPMDh, such as pH dependence, effects of divalent metal ions and inhibitors, temperature stability, and kinetic parameters, were determined for the first time *via* an enzyme coupling assay, wherein tAHMP prepared *via* stereoselective chemical synthesis (Yasuno et al., 2021) was employed as a substrate for the reverse reaction. Moreover, by using UV–visible spectrometry, EPR analysis, and iron quantification, we were able to demonstrate the existence of a [4Fe-4S] cluster in ApPMDh. The results from the mutagenetic study suggest three highly conserved cysteine residues as ligands for the [4Fe-4S] cluster and support the conclusion that PMDh exploits the cluster as a catalytic center, probably through a reaction mechanism similar to that proposed for aconitase superfamily enzymes.

## 2. Materials and methods

### 2.1. Materials

tAHMP was synthesized as previously described (Yasuno et al., 2021). Other reagents were purchased from Sigma-Aldrich (United States), Fujifilm Wako Pure Chemicals Co. (Osaka, Japan), or Kanto Chemical Co., Inc. (Tokyo, Japan).

### 2.2. Expression and purification of recombinant ApPMDh

The plasmid constructed in our previous study for the co-expression of the polyhistidine-tagged large subunit Ape_2087.1 and the untagged small subunit Ape_2089 in *E. coli* cells (Yasuno et al., 2021) was mutagenized to replace the thrombin-recognition sequence LeuValProArgGlySer, which exists between an N-terminal polyhistidine tag and Ape_2087.1, with the HRV3C-recognition sequence LeuGluValLeuPheGlnGlyPro. Mutagenesis was performed using a QuikChange II site-directed mutagenesis kit (Agilent, United States) with the primers 5’-atcacagcagcggcC TGGAAGTTCTGTTCCAG GGCCCGcatatgatgtacctg-3′ and its complementary primers. The capitalized region encodes the HRV3C-recognition sequence, which replaces the thrombin recognition sequence coded in the nucleotide sequence CTGGTGCCGCGCGGCAGC. The mutagenized plasmid was named pET-HisApPMDh.

The expression system of untagged Ape_2089 was constructed using an NcoI/BamHI-digested pET15b vector and an Ape_2089 gene fragment amplified from pET-HisApPMDh using KOD-Plus-Neo DNA polymerase and a pair of primers:5′-gaagtccatggaggtc ctcaggtacaggtcc-3′ and 5′-gaagtggatccttagggctggccagggcctagg-3.’ The underlined sequences of the primers are the recognition sites for NcoI and BamHI, respectively. The amplified fragment was inserted into the vector using Ligation High (TOYOBO, Osaka, Japan) to yield the plasmid pET-ApPMDh-S.

A strain of *E. coli*, Rosetta2 (DE3), was transformed with pET-HisApPMDh. The transformants were cultivated in 1 l Auto Induction medium supplemented with 100 μg/ml ampicillin and 30 μg/ml chloramphenicol at 37°C for 18 h. After harvesting by centrifugation, the cells were placed in an anaerobic glovebox, YUL-800A (UNICO, Tokyo, Japan), equipped with an OTE-70 deoxidizing catalyst unit. All manipulations for enzyme purification were performed anaerobically under 100% N_2_ gas using deoxygenated reagents. The cells were dissolved in a Histrap binding buffer containing 20 mM sodium phosphate (pH 7.4), 500 mM NaCl, and 20 mM imidazole and then disrupted using an ultrasonic homogenizer, THU-80 (AS ONE, Osaka, Japan), for six 1-min sessions of sonication. After centrifugation at 12,000 × *g* for 20 min, the supernatant was heat-treated at 60°C for 30 min. After centrifugation at 12,000 × *g* for 20 min, the supernatant was recovered and filtered using a syringe filter (0.45 μm pore size). The filtrate was applied to a 1-mL Histrap FF column (GE Healthcare, United States) equilibrated with Histrap binding buffer. After washing the column with 10 ml Histrap binding buffer, proteins bound to the column were eluted with Histrap elution buffer containing 20 mM sodium phosphate (pH 7.4), 500 mM NaCl, and 150 mM imidazole. The eluted protein was used as the purified ApPMDh. To estimate the ratio of untagged Ape_2089, the small subunit of ApPMDh co-purified with the tagged large subunit Ape_2087.1, the cells of *E. coli* Rosetta2 (DE3) transformed with pET-ApPMDh-S were cultivated and harvested in a manner similar to that of pET-HisApPMDh. Approximately equal amounts of the two cell types were mixed and subjected to the same affinity purification process as described above. The purification and subunit ratios of purified ApPMDh were checked using 15% SDS-PAGE and ImageJ software (Schneider et al., 2012).

### 2.3. Reconstruction of ApPMDh

Under anaerobic conditions, purified ApPMDh was incubated overnight at RT with 5 mM dithiothreitol. Subsequently, a 10-fold molar excess of FeCl_3_ was added to the enzyme solution. After 5 min, a molar amount of Na_2_S, equal to that of FeCl_3_ was added. After 2 h of incubation, unbound ions were removed from the ApPMDh using a PD-10 desalting column (GE Healthcare). ApPMDh was recovered from the column and used for analyses as reconstructed ApPMDh within 12 h after reconstruction. The UV–visible spectra of purified ApPMDh and reconstructed ApPMDh were measured anaerobically using a UV-2450 spectrophotometer (Shimadzu, Kyoto, Japan) and a quartz cuvette that could be tightly sealed with a rubber cap (Hellma Analytics, Müllheim, Germany). The UV–visible spectrum of oxidized ApPMDh, obtained by overnight exposure of the reconstructed ApPMDh to air, was also measured.

### 2.4. Enzyme-coupling PMDh assay

The activity of ApPMDh was quantitatively measured for the reverse reaction of tAHMP to MVA5P as described in our previous study (Yasuno et al., 2021), excepting that anaerobic PMDh reaction was carried out, in a 100-μL volume, at 70°C for 20 min (30 min for assay with inhibitors) in 70 mM Tris–HCl buffer, pH 7.0. The concentration of ApPMDh was controlled; for example, to 17 nM for purified ApPMDh or 5 nM for reconstructed ApPMDh, to match the initial rate wherein less than 10% of the substrate reacted. To elucidate the thermostability of ApPMDh, 5 μM of a reconstructed ApPMDh solution was treated for 30 min at 60, 70, 80, 90, or 100°C and used for the reaction. To investigate the effects of divalent cations or inhibitors, final 1 mM of a divalent cation (Mg^2+^, Mn^2+^, or Zn^2+^) or an inhibitor (iodoacetamide, H_2_O_2_, or EDTA) was added to the reaction mixture. To determine the pH dependence of ApPMDh, 70 mM PIPES-NaOH (pH 6.1–7.0), Tris–HCl (pH7.0–8.6), or CHES-NaOH (pH 8.5–9.6) was used instead of a buffer.

For the kinetic assay, we used a different method (Supplementary Figure 2). The anaerobically prepared reaction mixture of a 300 μL volume contained 0, 10, 15, 20, 30, or 50 μM tAHMP, 70 mM Tris–HCl buffer (pH 7.0 at 70°C), and 0.22 nM reconstructed ApPMDh, which was reconstructed on the same day. After incubation at 70°C for 20 min, the PMDh reaction was stopped on ice and removed from the anaerobic chamber. The enzyme was removed using a 0.5-mL AMICON ULTRA filter (10 kDa cutoff, Merck Millipore). In each well of a 96-well microtiter plate, 50 μL of the ultrafiltrate or standard MVA5P solution containing 0, 1.2, 2.0, 4.0, 16, or 32 μM of (*R,S*)-MVA5P (Sigma Aldrich) was mixed with 48 μL of a coupling enzyme solution, which contained 290 mM sodium phosphate, pH 7.5, 10 mM MgCl_2_, 420 μM Amplite™ ADHP (10-acetyl-3,7-dihydroxyphenoxazine, COSMO BIO Co., Ltd., Tokyo, Japan), 2.1 mM phosphoenolpyruvate, 1 unit of pyruvate kinase from rabbit muscle (Fujifilm Wako Pure Chemicals), 0.4 units of pyruvate oxidase from microorganism (TOYOBO), 1 unit of horse radish peroxidase (Oriental Yeast), and 3.7 μM of phosphomevalonate kinase from *Saccharolobus solfataricus*, which was prepared as described in our previous study (Nishimura et al., 2013) and confirmed not to react with tAHMP. After preheating at 37°C, the reaction was initiated by the addition of a 2-μL solution of 10 mM ATP. During the reaction at 37°C, the absorbance at 571 nm was monitored every minute, after 3 s of shaking, using an Infinite 200 PRO microplate spectrophotometer (TECAN, Männedorf, Switzerland). Spectrometric analysis of each sample was performed in quadruplicate. The amount of (*R*)-MVA5P in the ultrafiltate samples was quantified using a standard curve based on the data from the standard solutions. Kinetic parameters were determined by Michaelis–Menten curve fitting to the substrate concentration vs. initial velocity plot using GraphPad Prism 9 (GraphPad Software, United States).

### 2.5. Site-directed mutagenesis of ApPMDh

Plasmids for the expression of mutated enzymes were constructed using the QuikChange II site-directed mutagenesis kit (Agilent) using the plasmids pET-HisApPMDh, KOD-Plus-Neo DNA polymerase (TOYOBO) and the primers listed below (one of the complementary primer pairs is shown): for C122A, 5’-gacttgatactgacg GCca cgccctactacacc-3′; for E145A, 5’-gtggcctggggcgCgtcc agcgcagtg-3′; for C297A, 5’-cctctacgttggcGCcccccacgccagcatag-3′; and for C356A, 5’-cgtgagggactccGCcctcatagtcagcccc-3.’ Capitalized letters indicate mismatched nucleotides. Recombinant expression, purification, UV–visible spectrum measurement, and assays of mutated enzymes were performed using the same procedures as those described above for wild-type ApPMDh.

### 2.6. EPR analysis of ApPMDh

Under anaerobic conditions, the buffer of the purified ApPMDh solution was exchanged with HRV3C protease buffer containing 20 mM Tris–HCl (pH 7.9), 150 mM NaCl, and 10% glycerol. The polyhistidine tag fused with purified ApPMDh was then cleaved by adding 10 units of HRV3C protease (Funakoshi, Tokyo, Japan) per 1 mg purified ApPMDh, followed by incubation with shaking at 4°C for 24 h. The reaction mixture was loaded into a 1-mL Histrap FF column (GE Healthcare) equilibrated with HRV3C protease buffer, and the flow-through fraction was recovered. The fraction contained tag-free ApPMDh, from which the polyhistidine tag was removed. The enzyme was reconstructed with FeCl_3_ and Na_2_S, as described above, with the exception of 100 mM glycine-KOH buffer (pH 10.0) containing 20 mM imidazole and 20% glycerol in the desalting treatment of the enzyme in a PD-10 column. The tag-free ApPMDh was concentrated using a 0.5-mL AMICON ULTRA filter (10 kDa cutoff, Merck Millipore) until its concentration exceeded 10 mg/ml (180 μM). A 50-fold molar excess of Na_2_S_2_O_4_ was then added to the enzyme solution, followed by incubation at 40°C for 30 min to prepare reduced ApPMDh. A nonreduced ApPMDh sample was prepared using the same method, without Na_2_S_2_O_4_. A substrate-binding ApPMDh sample was prepared by adding a three-fold molar excess of (*R,S*)-MVA5P to the reduced ApPMDh sample.

In the anaerobic chamber, 150 μL of each sample was placed into a Suprasil quartz EPR tube (5 mm OD) and frozen in liquid nitrogen. The frozen sample tube was sealed, removed from the chamber, and analyzed using an E580 EPR spectrometer (Bruker, United States) equipped with an ER 4122SHQ cavity and ESR 910 cryostat (Oxford Instruments, Abingdon-on-Thames, GB). The measurement conditions were as follows: microwave frequency, 9.48 GHz; microwave power, 10 mW; center field, 3,456 G; modulation amplitude, 18 G; modulation frequency, 100 kHz; time constant, 163 ms; sweep time, 167 s.

Data fitting of the micropower dependence of the EPR signal intensity was performed following Equation 1 (Rupp et al., 1978), where *P* and *b* represent microwave power and inhomogeneous parameters, respectively.


(Equation 1) $$Intensity\propto \frac{P^{0.5}}{{\left[1+P/P_{1/2}\right]}^{0.5b}}$$

### 2.7. Iron assay of ApPMDh

The reconstruction of tag-free ApPMDh was performed as described above, except that Li_2_S, which is less hygroscopic than Na_2_S (Freibert et al., 2018), was used in the Fe-S cluster reconstruction process. The protein concentration in the enzyme solution was measured using the Bradford method with a Protein Assay CBB Solution (Nacalai Tesque, Inc., Kyoto, Japan). The enzyme solution was diluted 10 times with deionized water, after which HCl was mixed to a final concentration of 0.06 M. The concentration of iron in the sample solution was measured based on the absorbance of chelating complex of Fe^2+^ and 2-nitroso-5-[*N*-n-propyl-*N*-(3-sulfopropyl)amino]phenol using an iron assay kit LS (Metallogenics Co., Ltd., Chiba, Japan) and compared with standard iron solutions. Absorbance at 560 nm was measured using an Infinite 200 PRO microplate spectrophotometer (TECAN).

### 2.8. Construction of ApPMDh model structure

The structural model of apo-ApPMDh, which consists of only the large and small subunit proteins, was constructed with Colabfold2 (Mirdita et al., 2022) working on Google Colaboratory, and the [4Fe-4S] cluster and MVA5P were manually added based on the aligned structure of putative TkPMDh (pdb#:7CNS). The holo-ApPMDh model was optimized by simplified molecular dynamics calculations (without solvation) using Molby (Nagata, 2014), which integrated AmberTools (Case et al., 2005). Visualization, editing, and figure construction of structural data were performed using Open-source Pymol (Schrödenger, LLC, United States).

## 3. Results

### 3.1. Reconstituting the catalytic center of ApPMDh

**Figure 3:**
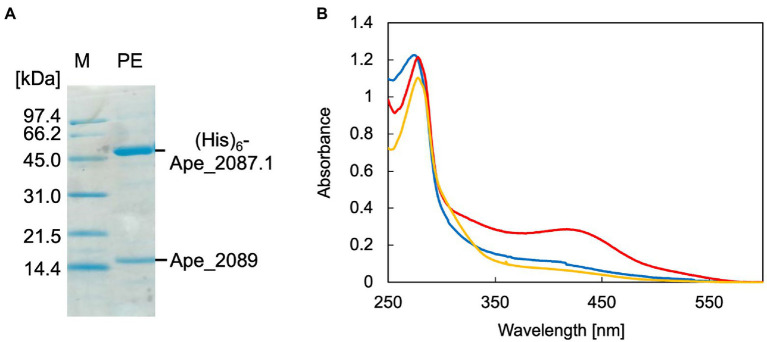
SDS-PAGE and UV–visible spectra of *Aeropyrum pernix* phosphomevalonate dehydratase (ApPMDh). **(A)** SDS-PAGE of purified ApPMDh containing a polyhistidine-tagged large subunit, (His)_6_-Ape2087.1, and a small subunit without tag, Ape_2089. M, molecular marker proteins; PE, purified enzyme. **(B)** UV–visible spectra of ApPMDh before (blue) and after reconstruction (red) or after air-oxidation (yellow).

Recombinant ApPMDh was expressed in *E. coli* cells for characterization. The large subunit, Ape_2087.1, of ApPMDh fused with an N-terminal polyhistidine tag was co-expressed with the small subunit Ape_2089 with no affinity tag sequence in the same bacterial cells. Applying affinity chromatography with a polyhistidine tag enabled the purification of both the large and small subunits, revealing the formation of a heteromeric complex. Based on the protein bands detected by SDS-PAGE analysis, the ratio of the subunits appeared to be 1:1 (Figure 3A). For confirmation, ApPMDh was purified in a similar manner from a mixture of cells expressing both polyhistidine-tagged Ape_2087.1 and tag-free Ape_2089, and those expressing only tag-free Ape_2089 (Supplementary Figure 3A). An intense band for tag-free Ape_2089 was observed in the cell-free protein sample that was used for affinity purification, indicating that an excess amount of the tag-free small subunit was supplied. However, after purification, the ratio of the subunits remained approximately 1:1, and most of the Ape_2089 had become part of the flow-through fraction. Based on these observations, we conclude that ApPMDh is a heteromultimeric protein composed of the same number of large and small subunits, similar to other members of the aconitase superfamily.

Biochemical characterization was performed using purified ApPMDh. Because the brown color of ApPMDh purified from aerobically grown *E. coli* cells was sometimes very pale and diminished after oxidation, we assumed that most of the purified enzyme could be in the apo state, lacking the cofactor in its catalytic center, but the nature of the colored cofactor was unclear at that time. This is in contrast to the homolog from *T. kodakarensis*, which was reported to have a brown color even after crystallization (Watanabe et al., 2021). Thus, we attempted to reconstruct the catalytic center using a standard protocol for iron–sulfur cluster reconstruction. Anaerobic treatment of purified ApPMDh with excess FeCl_3_ and then with Na_2_S, followed by desalting, yielded an enzyme solution with a deeper brown color. The UV–visible spectrum of the reconstructed ApPMDh showed an absorption maximum at 410 nm, which could be attributed to the existence of an unidentified cofactor (Figure 3B). Before and after the reconstruction process, the ratio of absorbance at 410 and 280 nm (A_410_/A_280_) for purified ApPMDh was 9.5 and 23.8%, respectively. After overnight air exposure of the reconstructed ApPMDh solution, the solution turned colorless and A_410_/A_280_ decreased to 6.3%. Based on the supposition that the reconstructed ApPMDh was in a complete holo-state and the air-oxidized ApPMDh was in a complete apo-state, calculations suggested that only approximately 18% of the purified ApPMDh was bound to the colored cofactor.

Subsequently, we measured the activity of purified, reconstructed, and air-oxidized enzymes using a recently developed enzyme-coupling PMDh assay method (Yasuno et al., 2021). In this assay, synthetic tAHMP was used as a substrate for the reverse reaction of PMDh performed anaerobically at 70°C. Then MVA5P formed by the reaction was measured by coupling with the oxidation of NADH. Using this method, the specific activities of the purified and reconstructed ApPMDh were determined to be 2.86 ± 0.3 and 14.8 ± 2.2 μmol/min/mg, respectively, and the air-oxidized ApPMDh was completely inactive. This indicated that the enzyme was activated by the reconstruction process. The activity of the reconstructed ApPMDh was five times higher than that of the purified enzyme, which is in good agreement with the above-calculated percentage of holo-enzyme in the purified ApPMDh.

### 3.2. Biochemical characterization of ApPMDh

**Figure 4:**
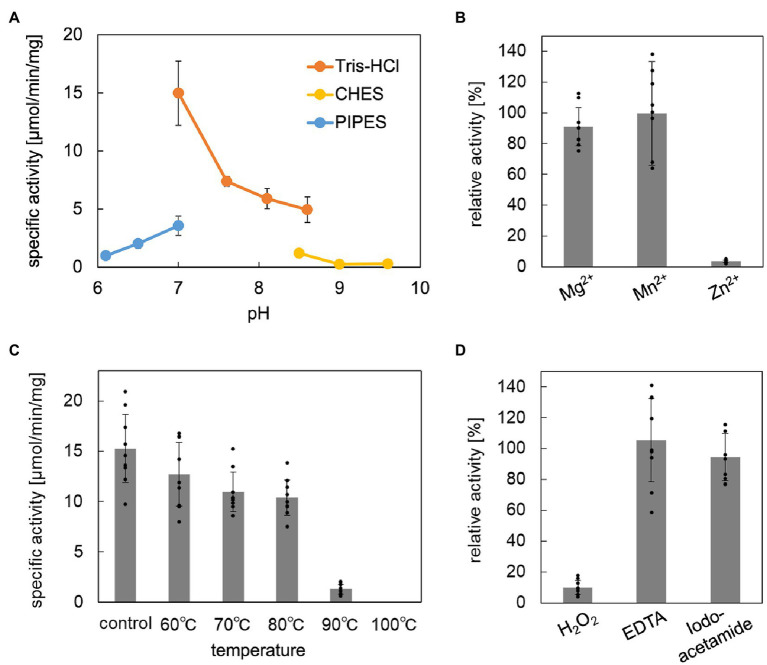
Biochemical properties of *Aeropyrum pernix* phosphomevalonate dehydratase (ApPMDh). **(A)** pH dependence. **(B)** Effect of metal ion at 1 mM each. **(C)** Thermostability after 30 min of heat treatment. **(D)** Effects with 1 mM additives. ApPMDh reaction using *trans*-anhydromevalonate phosphate (tAHMP) as a substrate was conducted anaerobically at 70°C for 20 min for **(A–C)** and 30 min for **(D)**. All reactions were performed in triplicate. The amount of produced MVA5P in each reaction mixture, from which ApPMDh had been removed by ultrafiltration, was colorimetrically analyzed in triplicate through the coupling reactions of phosphomevalonate decarboxylase, pyruvate kinase, and lactate dehydrogenase, which caused a commensurate decrease in NADH, as measured by absorbance at 340 nm. The amount of (*R*)-mevalonate 5-phosphate (MVA5P) was quantified based on a standard curve. Error bars indicate standard deviations. For **(B,D)**, the activity without additive was set at 100%.

The enzymatic properties, such as pH dependence, effects of metal ions, thermostability, and tolerance against chemical inhibitors, of the reconstructed ApPMDh were analyzed to compare the enzyme with other known aconitase superfamily members. The optimal pH for ApPMDh was 7.0, as shown in Figure 4A. Neither the addition of 1 mM Mg^2+^ nor 1 mM Mn^2+^ had a significant effect on the enzyme activity, whereas Zn^2+^ had an inhibitory effect on the ApPMDh reaction, causing almost complete inactivation (Figure 4B). Notably, inhibition by Zn^2+^ has been reported for both bacterial type I AcnX and mammalian mitochondrial aconitase (Costello et al., 1997; Watanabe et al., 2016), although we cannot exclude the possibility that Zn^2+^ inhibits the other enzymes utilized in the coupling assay. Thermostability of ApPMDh was analyzed by applying 30 min of heat treatment to an enzyme solution at temperatures ranging from 60 to 100°C, followed by enzyme assay at 70°C. After treatment at 60–80°C, the activity of ApPMDh was reduced, but not at a significant level compared with that of the enzyme without heat treatment. Treatment at 90°C, however, caused a loss of most enzyme activity, and treatment at 100°C resulted in complete inactivation (Figure 4C). Considering the optimal growth temperature of *A. pernix* at 90–95°C, the observed thermostability of ApPMDh seemed somewhat lower, although this was likely because of the *in vitro* conditions of the heat treatment. The enzyme assay, in the presence of potential chemical inhibitors, demonstrated that ApPMDh was strongly inhibited by H_2_O_2_, which agrees with the reported sensitivity of aconitase toward reactive species (Castro et al., 2019; Figure 4D). In contrast, neither EDTA nor iodoacetamide showed significant inhibitory effects. The insensitivity toward the chelating agent EDTA is in good agreement with the finding described above, wherein the addition of Mg^2+^ or Mn^2+^ did not enhance ApPMDh activity, suggesting that ApPMDh does not require divalent cations to bind its phosphorylated substrates, MVA5P and tAHMP, and to catalyze dehydration/hydration. Although the insensitivity toward the thiol modification agent iodoacetamide appears to contradict the expected catalytic importance of conserved cysteine residues in PMDh, mitochondrial aconitase has also been reported to only be slightly inhibited by EDTA and iodoacetamide, probably suggesting the tolerance of its [4Fe-4S] cluster to such reagents (Kennedy et al., 1983, 1988).

To perform a kinetic study of ApPMDh, we developed a different enzyme-coupling assay method that enabled high sensitivity and low background detection of the PMDh reaction with a lower concentration of tAHMP. In this method, the concentration of MVA5P formed from tAHMP by the anaerobic PMDh reaction at 70°C was measured afterwards by the formation of a dye, resorufin, from anaerobic enzyme-coupling reactions of phosphomevalonate kinase, pyruvate kinase, pyruvate oxidase, and peroxidase (Supplementary Figure 2). Using the new assay method, we found the *K*_m_ and *k*_cat_ of the reconstructed ApPMDh to be 18.3 μM and 15.2 s^−1^, respectively (Supplementary Figure 4). The *k*_cat_/*K*_m_ value, 8.28 × 10^5^ M^−1^·sec^−1^, was larger than that of type I AcnX from *A. tumefaciens* in the reaction with *cis*-3-hydroxy-L-proline (2.87 × 10^4^ M^−1^· sec^−1^; Watanabe et al., 2021), whereas the activity of PMDh was measured with the reverse reaction from tAHMP to MVA5P. It should be also noted that ApPMDh assay was performed at the temperature that was unlikely optimal for the enzyme from the hyperthermophilic archaeon.

### 3.3. Effect of mutagenesis on the catalytic center of ApPMDh

Site-directed mutagenesis was performed on the conserved residues that are supposedly involved in cofactor binding at the catalytic center of ApPMDh: Cys122 corresponds to site A of the AcnX family enzymes; Cys297 corresponds to site 1 of most aconitase superfamily enzymes; and Cys356 corresponds to site B of the AcnX family proteins and site 2 of most aconitase superfamily enzymes (Figure 2). Additionally, Glu145, which is highly conserved among all AcnX family enzymes, was selected for mutagenesis, given that alanine replacement of the corresponding residue was reported to cause complete inactivation of *P. aeruginosa* type I AcnX (Watanabe et al., 2016). Indeed, the corresponding glutamate residue interacts with the water molecule coordinated to the [2Fe-2S] cluster in the crystal structure of *A. tumefaciens* type-I AcnX (pdb#:7CNP). Alanine replacement of each residue was performed to construct the mutant enzymes C122A, E145A, C297A, and C356A (Figure 2). Affinity purification of the mutants yielded purified proteins with similar levels of purity, except for C297A, which contained faint but not negligible bands of smaller (<14.4 kDa) proteins that were likely derived from its partial degradation (Supplementary Figure 3B). However, reconstruction of the mutants resulted in different A_410_/A_280_ ratios (Supplementary Figure 5A). The A_410_/A_280_ ratios of C122A, C297A, and C356A (7.5, 8.1, and 10.7%, respectively) were much lower than those of the reconstructed wild-type ApPMDh (23.8%) and more closely approximated that of the air-oxidized ApPMDh (6.3%). An enzyme-coupled assay of the mutants demonstrated that the A_410_/A_280_ ratio was in good agreement with the PMDh activity. The E145A mutant retained 76% of the activity of the wild-type ApPMDh, whereas the activity of the other mutants was less than 4% of that of the wild type (Supplementary Figure 5B). These results suggest that the three conserved cysteine residues of ApPMDh (Cys122, Cys297, and Cys356) are involved in the formation of one iron–sulfur cluster in the enzyme, whereas Glu145 probably has no direct role in cofactor formation. Although our mutagenic study was performed before the publication of the crystal structures of TkPMDh (pdb#:7CNR and 7CNS; Watanabe et al., 2021), the structure demonstrated that in the putative PMDh, the corresponding three cysteine residues actually coordinate a [3Fe-4S] cluster. However, the [3Fe-4S] cluster does not fit well with the dehydration/hydration-catalyzing activity of PMDh, as also suggested by the authors. Similar to ordinary aconitase superfamily enzymes, a [4Fe-4S] cluster, which can provide vacant iron for catalysis, appears to be a more appropriate cofactor of PMDh.

### 3.4. EPR analysis of ApPMDh

**Figure 5:**
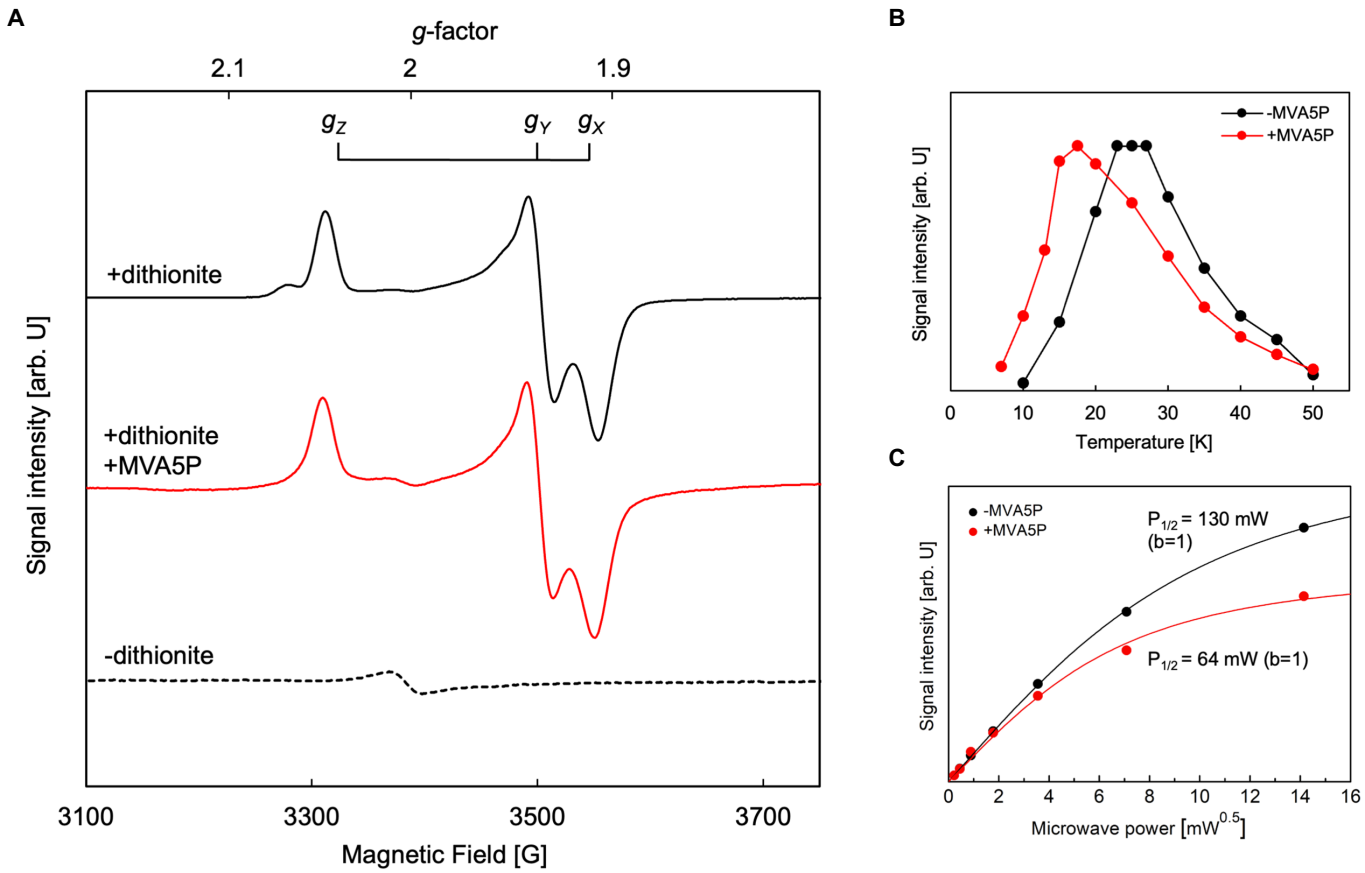
Electron paramagnetic resonance (EPR) analysis of *Aeropyrum pernix* phosphomevalonate dehydratase (ApPMDh). **(A)** EPR spectra of untreated ApPMDh at 25 K (broken line), dithionite-treated ApPMDh at 19 K (black line), and dithionite-treated ApPMDh in the presence of MVA5P at 17.5 K (red line). Measurements were performed at microwave frequency of 9.48 GHz. **(B)** Temperature dependence and **(C)** microwave power dependence at 25 K of the EPR signal intensity of dithionite-treated ApPMDh. Data in the presence and absence of (*R*)-mevalonate 5-phosphate (MVA5P) are shown in red and black, respectively. Lines in **(C)** are the fitting curves based on Equation 1 in the Materials and Methods section.

EPR analysis was performed to identify the type and structure of the catalytic center of active ApPMDh. To exclude the possibility of artifactual metal ion binding, the polyhistidine tag was removed from purified ApPMDh (Supplementary Figure 3B). Tag-free ApPMDh was subjected for iron–sulfur cluster reconstruction in glycine-KOH buffer, pH10. UV–visible spectrum of the reconstructed tag-free ApPMDh had the value of A_410_/A_280_ with 25.5%, suggesting the formation of the same iron–sulfur cluster with that in the reconstructed tagged-ApPMDh (Supplementary Figure 6). After concentration, the enzyme was treated with or without excess sodium dithionite and analyzed by EPR spectroscopy. We confirmed that reconstructed tag-free ApPMDh had activity of 1.00 ± 0.17 μmol/min/mg at pH10, which was comparable with 0.30 ± 0.17 μmol/min/mg of the reconstructed tagged ApPMDh in CHES buffer, pH9.6 (Figure 4A). In Figure 5A, we present the observed EPR spectra of dithionite-treated (top) and untreated (bottom) ApPMDh. The spectrum of dithionite-treated ApPMDh had clear rhombic-type EPR signals with *g_Z_* = 2.04, *g_Y_* = 1.94, and *g_X_* = 1.91, whereas the untreated enzyme was almost EPR-silent and gave only a faint signal at *g* = ~2.0. The shape of the spectrum of dithionite-treated ApPMDh was similar to that reported for bovine mitochondrial aconitase (Emptage et al., 1983). The signals of dithionite-treated ApPMDh were detectable only at extremely low temperatures, ranging from 10 to 50 K, with the maximum strength obtained at approximately 25 K (Figure 5B). These properties of the spectrum, that is, its rhombic shape, appearance only after dithionite reduction, and dependence on extremely low temperatures, strongly suggest that ApPMDh has a [4Fe-4S]-type iron–sulfur cluster, rather than a [3Fe-4S] or [2Fe-2S] cluster (Lancaster et al., 1979; Beinert et al., 1996). Indeed, the EPR spectrum of dithionite-treated ApPMDh, which was presumably derived from an S = 1/2 ground spin state [4Fe-4S]^1+^, was distinct from the reported EPR signal of dithionite-treated *cis*-3-hydroxy-L-proline dehydratase (type I AcnX) from *A. tumefaciens*, which had *g*-values of 2.02, 1.94, and 1.86, attributed to the [2Fe-2S] cluster (Watanabe et al., 2021).

The EPR spectrum of dithionite-treated bovine mitochondrial aconitase was reported to change in shape when citrate, a substrate of the enzyme, was added (Emptage et al., 1983). The *g*-values without the substrate were 2.06, 1.93, and 1.86, but when 0.1 mM citrate was added, the *g*-values shifted to 2.04, 1.85, and 1.78, respectively. This change in the EPR signal shape, along with data from other spectroscopic observations, such as Mössbauer and ENDOR, was attributed to the coordination of the substrate to the [4Fe-4S] cluster (Beinert et al., 1996). The coordination of a substrate, isocitrate or citrate, to a vacant iron in the [4Fe-4S] cluster of aconitase was clearly demonstrated by the crystal structures of mitochondrial aconitases from porcine and bovine sources (pdb#:7ACN, 1C96, and 1C97), in which the other three irons of the cluster were coordinated to three conserved Cys residues (Lauble et al., 1992; Beinert et al., 1996). Thus, we added approximately 0.5 mM of (*R,S*)-MVA5P, a substrate of ApPMDh, to an enzyme solution prepared as described above and performed EPR analysis after dithionite treatment. However, the observed EPR spectrum (Figure 5A, middle) was almost identical to that observed in the absence of the substrate (upper). This phenomenon implies that MVA5P might not bind to the catalytic center of ApPMDh when the iron–sulfur cluster is reduced to [4Fe-4S]^1+^, possibly because [4Fe-4S]^2+^ is the catalytically active species. However, the temperature dependence of the EPR spectrum differed in the absence and presence of the substrate (Figure 5B). The maximum EPR signal intensity was obtained at approximately 25 K in the absence of MVA5P, and the temperature of the maximum intensity decreased to 17.5 K when the substrate was added. The microwave power dependence of the EPR signal intensity also changed between dithionite-treated ApPMDh with and without MVA5P (Figure 5C). The *P*_1/2_ value decreased from 130 mW to 65 mW, with the inhomogeneous parameter *b* of 1, with the addition of the substrate. These differences are likely indicative of the binding of the substrate near the cluster, but the similarity of the EPR spectra shapes suggests that the binding mode of MVA5P in ApPMDh could be different from that of citrate in aconitase and, therefore, would not significantly affect the structure and/or charge distribution of the [4Fe-4S] cluster.

### 3.5. Quantification of iron in ApPMDh

The enzyme was desalted and concentrated in an anaerobic chamber immediately after reconstruction of the iron–sulfur cluster of tag-free ApPMDh. The protein concentration of the enzyme solution was found to be 21.1 ± 1.6 μM. The solution was diluted and acidified with HCl, and the Fe^2+^ concentration was measured based on the formation of a colorimetric chelating complex after the reduction of iron ions. The iron concentration in the ApPMDh solution before dilution was determined to be 86.8 ± 2.6 μM. Therefore, the number of iron atoms contained in an ApPMDh molecule (the complex of the large and small subunits) was calculated to be 4.11. From both the results and EPR data, we concluded that a [4Fe-4S]-type iron–sulfur cluster exists in ApPMDh.

## 4. Discussion

**Figure 6:**
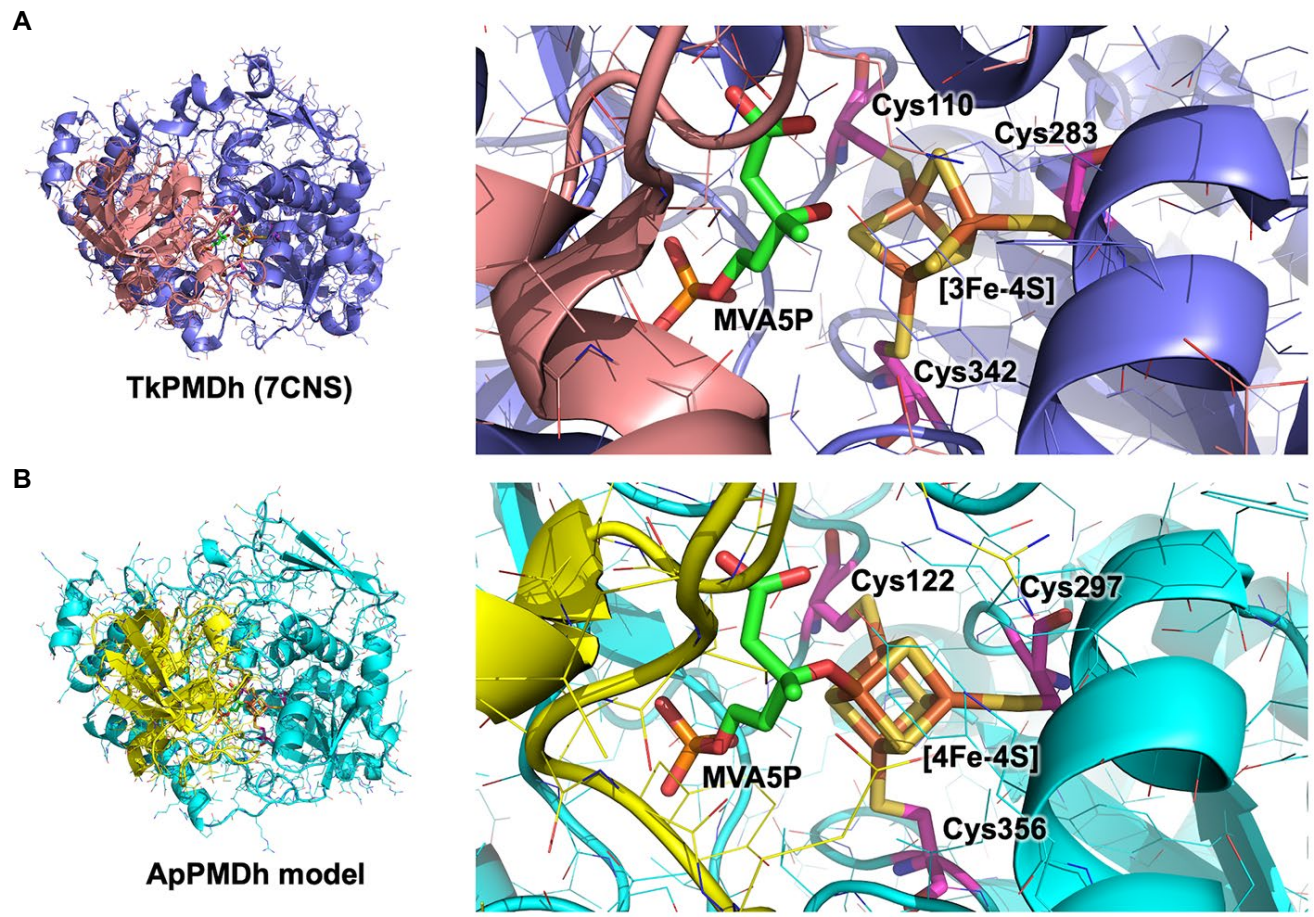
Putative catalytic center structure of *Aeropyrum pernix* phosphomevalonate dehydratase (ApPMDh). The structures of the overall protein complex (left) and the catalytic center containing an iron–sulfur cluster (right) of *Thermococcus kodakarensis* phosphomevalonate dehydratase (TkPMDh) [**(A)**, pdb#: 7CNS] are shown, along with those of modeled ApPMDh **(B)**. The large and small subunits of TkPMDh are colored in dark blue and orange, and those of ApPMDh are in cyan and yellow, respectively. (*R*)-mevalonate 5-phosphate (MVA5P) and cysteine residues coordinating the iron–sulfur cluster are represented in stick models in green and magenta, respectively. The structure model of apo-ApPMDh was constructed using Colabfold (Mirdita et al., 2022), after which the [4Fe-4S] cluster and MVA5P were manually added based on the aligned structure of putative TkPMDh (pdb#: 7CNS). The model of holo-ApPMDh was optimized *via* simplified molecular dynamics using Molby (Nagata, 2014).

In the present study, we revealed the enzymatic properties of an archaeal AcnX family enzyme, ApPMDh, which plays a key role in the recently discovered archaeal MVA pathway. Our results demonstrate that ApPMDh harbors a [4Fe-4S] cluster, similar to most hydratases/dehydratases belonging to the aconitase superfamily, whereas the previously reported crystal structures of TkPMDh contain a [3Fe-4S] cluster (Watanabe et al., 2021). Considering the fact that the [4Fe-4S] cluster of mitochondrial aconitase is known to be converted into a [3Fe-4S] cluster through oxidation (Lauble et al., 1992; Beinert et al., 1996), the [3Fe-4S] cluster observed *via* structural analysis of TkPMDh might also be derived from its original active form, the [4Fe-4S] cluster, as speculated by Watanabe et al. (2021). The existence of the [4Fe-4S] cluster is consistent with the oxygen- and H_2_O_2_-sensitivity of ApPMDh, which is distinct from the reported oxygen tolerance of the bacterial homolog and similar to the oxygen sensitivity of the usual aconitase-superfamily hydratases/dehydratases. The tolerance of ApPMDh to EDTA and iodoacetamide also resembles the reported insensitivity of aconitase toward these agents (Kennedy et al., 1983, 1988). Based on our finding that the [4Fe-4S] cluster is required for enzyme activity, the catalytic role of the iron–sulfur cluster in ApPMDh is thought to be similar to that of aconitase. If so, the vacant iron of the cluster, which is not bound to conserved cysteine residues unlike the other irons in the cluster, is thought to be coordinated to the tertiary hydroxyl group of MVA5P in the enzyme-substrate complex to catalyze its elimination or to a water molecule as a second substrate to catalyze the transfer of a hydroxyl group to tAHMP in the reverse reaction. Indeed, the MVA5P-complex structure of TkPMDh (pdb#:7CNS) recently reported by Watanabe et al. (2021) suggests that the substrate is bound in the proximity of the [3Fe-4S] cluster with an appropriate conformation to undergo dehydration if additional iron exists (Figure 6A). The EPR spectra of ApPMDh, however, did not change with the addition of the substrate MVA5P, unlike the spectra of mitochondrial aconitase, which were largely affected by the addition of citrate (Emptage et al., 1983). This indicates that the electronic structure of the substrate-coordinating cluster in ApPMDh is similar to that of the substrate-free cluster, which is likely coordinated by a water molecule at the vacant iron. The MVA5P-complex structure of TkPMDh may explain this phenomenon. In the structure, neither the carboxyl group nor the phosphate group of MVA5P faces the [3Fe-4S] cluster because they are in contact with the surrounding residues, that is, Thr63, Ser130, and Lys361 for the carboxyl group and Gly48, Val49, Ser50, Asn53, Asn79, and Pro80 for the phosphate group. Thus, these ionized groups are unlikely to coordinate directly to the [4Fe-4S] cluster in the putative active form of the enzyme. In contrast, in both the citrate complex and isocitrate-complex structures of mammalian mitochondrial aconitase, a carboxyl group of the substrates was reported to interact with the vacant iron of the [4Fe-4S] cluster, as well as the hydroxyl group that would be eliminated (Lauble et al., 1992; Beinert et al., 1996). The interaction of the carboxyl group probably affects the electronic structure of the cluster, which would result in a significant change in the EPR spectra (Emptage et al., 1983). Based on these structural insights, we constructed a structural model of ApPMDh, whose large and small subunits have 38 and 44% sequence identities with those of TkPMDh, respectively (Figure 6B). In the modeled structure, three irons in the [4Fe-4S] cluster were coordinated by three cysteine residues, Cys122, Cys 297, and Cys356, and only the 3-hydroxyl group, not the carboxyl or phosphate group, of MVA5P was coordinated to the remaining iron.

Because ApPMDh, a type IIb AcnX, has a [4Fe-4S] cluster similar to other aconitase superfamily enzymes, it is surprising that its bacterial homolog, type I AcnX, uses a [2Fe-2S] cluster for the catalysis of hydroxyproline dehydration. Bacterial type I AcnX enzymes have been reported to be tolerant to oxidation (Watanabe et al., 2016), and this property might be attributed to the presence of the unusual [2Fe-2S] cluster. It remains unclear which type of iron–sulfur cluster exists in members of the other AcnX subfamilies. If a PMDh that utilizes a [2Fe-2S] cluster for catalysis is discovered or synthesized, it might be used for the bioproduction of isoprenoids through the ATP-saving archaeal MVA pathway (Hayakawa et al., 2018), as the existing archaeal MVA pathway is functional only under nearly anaerobic conditions when reconstructed in bacterial cells (Yoshida et al., 2020).

## Data availability statement

The raw data supporting the conclusions of this article will be made available by the authors, without undue reservation.

## Author contributions

MK and HH were involved in the study design, data analysis, and drafting of the article. MK, KK, HM, TI, and HH were involved in data acquisition. YY and TS provided reagents. All authors revised the manuscript, approved the manuscript for publication, and agreed to be accountable for all aspects of the work in ensuring that questions related to the accuracy or integrity of any part of the work are appropriately investigated and resolved.

## Funding

This work was supported by JSPS KAKENHI Grant Numbers 18 K19170, 19H04651 and 20H02899, and by grants-in-aid from the Institute for Fermentation, Osaka, the Noda Institute for Scientific Research, and the Nagase Scientific Technology Foundation for HH.

## Conflict of interest

The authors declare that the research was conducted in the absence of any commercial or financial relationships that could be construed as a potential conflict of interest.

## Publisher’s note

All claims expressed in this article are solely those of the authors and do not necessarily represent those of their affiliated organizations, or those of the publisher, the editors and the reviewers. Any product that may be evaluated in this article, or claim that may be made by its manufacturer, is not guaranteed or endorsed by the publisher.

## Supplementary material

The Supplementary material for this article can be found online at: https://www.frontiersin.org/articles/10.3389/fmicb.2023.1150353/full#supplementary-material
