# Supplementary material for: A [4Fe-4S] cluster resides at the active center of phosphomevalonate dehydratase, a key enzyme in the archaeal modified mevalonate pathway
Source: Front Microbiol. 2023 Mar 13;14:1150353. doi: 10.3389/fmicb.2023.1150353 (PMC10040528; doi:10.3389/fmicb.2023.1150353)
Supplement: Supplementary file 1 [file Data_Sheet_1.pdf]

*Supplementary Material*

# A [4Fe-4S] cluster resides at the active center of phosphomevalonate dehydratase, a key enzyme in the archaeal modified mevalonate pathway

**Mutsumi Komeyama[1], Kohsuke Kanno[1], Hiroyuki Mino[2], Yoko Yasuno[3], Tetsuro Shinada[3], Tomokazu Ito[1], and Hisashi Hemmi[1,*]**

[1]Graduate School of Bioagricultural Sciences, Nagoya University, Furo-cho, Chikusa-ku, Nagoya, Aichi, Japan

[2]Graduate School of Science, Nagoya University, Furo-cho, Chikusa-ku, Nagoya, Aichi, Japan

[3]Graduate School of Science, Osaka Metropolitan University, Sugimoto, Sumiyoshi, Osaka, Japan

*** Correspondence:**

Hisashi Hemmi

hhemmi@agr.nagoya-u.ac.jp

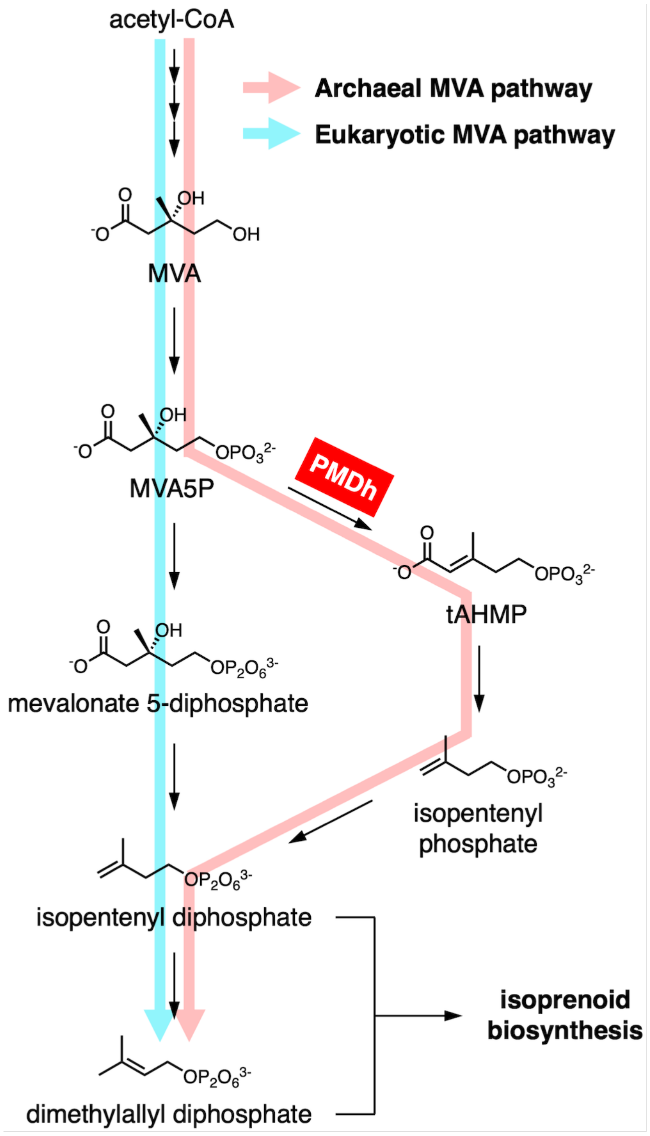


**Supplementary Figure 1.** Archaeal and eukaryotic mevalonate (MVA) pathways. MVA5P, mevalonate 5-phosphate; tAHMP, *trans*-anhydromevalonate 5-phosphate.

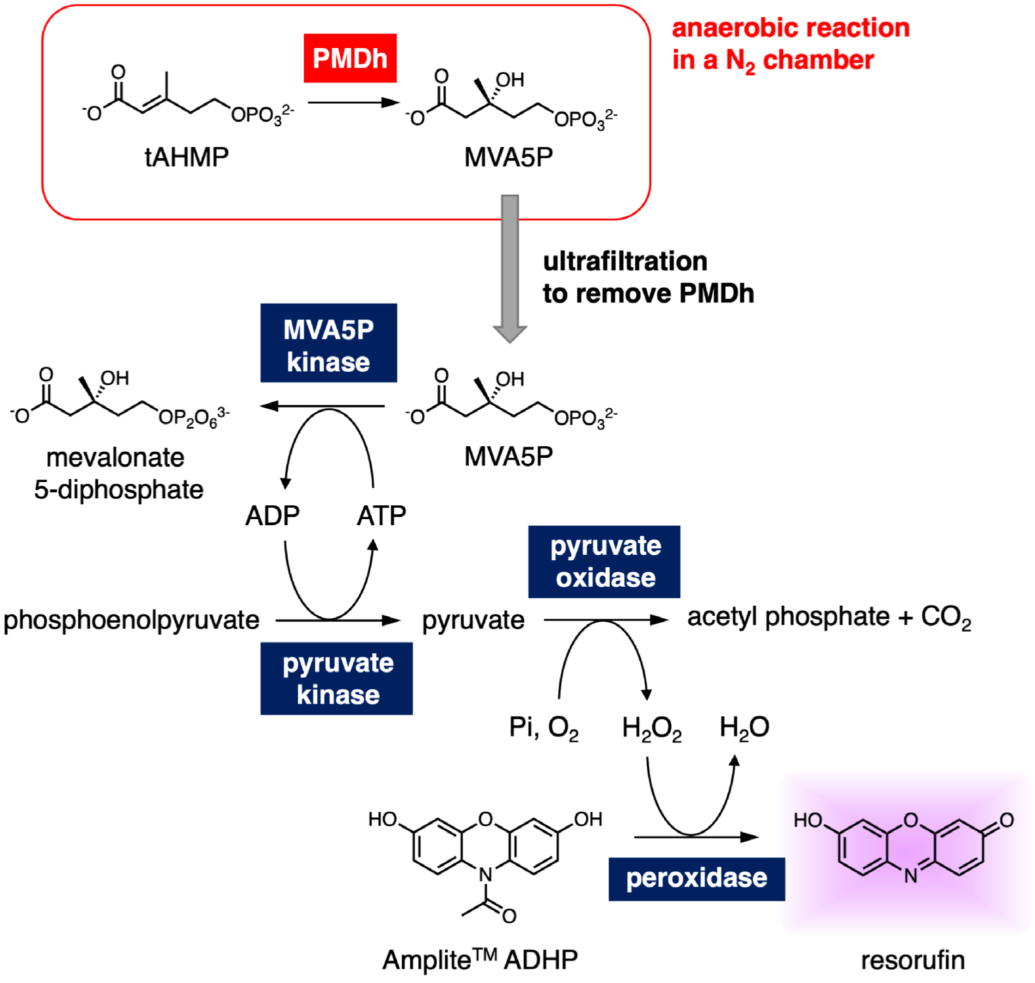


**Supplementary Figure 2.** Scheme of newly developed phosphomevalonate (PMDh) assay method used for kinetic analysis. MVA5P, mevalonate 5-phosphate; tAHMP, *trans*-anhydromevalonate 5-phosphate; Pi, inorganic phosphate.

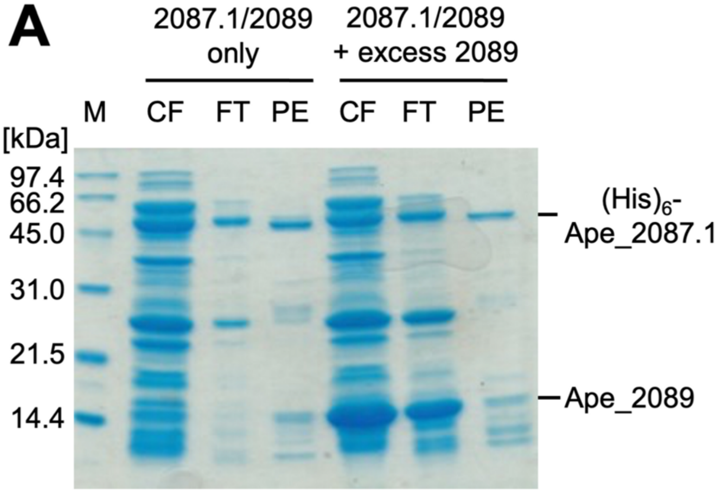


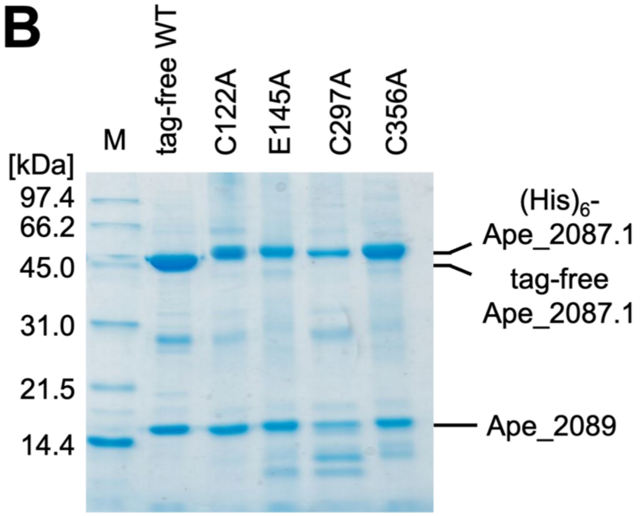


**Supplementary Figure 3.** SDS-PAGE of *Aeropyrum pernix* phosphomevalonate dehydratase (ApPMDh). (A) Confirmation of subunit ratios of ApPMDh (2087.1/2089) purified with or without the addition of excess Ape_2089 (2089). CF, cell-free protein fraction; FT, flow-through fraction. (B) Purified tag-free wild-type ApPMDh (WT) and polyhistidine-tagged ApPMDh mutants.

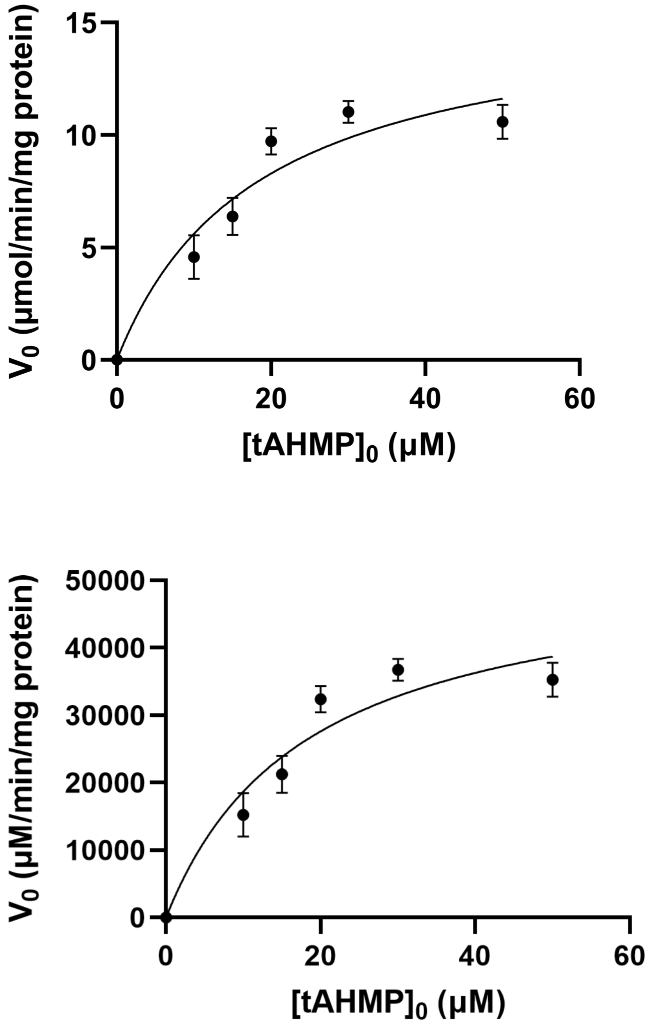


**Supplementary Figure 4.** Steady-state kinetic analysis of *Aeropyrum pernix* phosphomevalonate dehydratase (ApPMDh). The same data were represented using the unit of µmol/min/mg protein (upper panel) and µM/min/mg protein (lower panel) for y-axis. Data points and error bars indicate means and standard deviations of quadruple measurements, respectively. The smooth line represents a Michaelis–Menten curve fitted to the measured data.

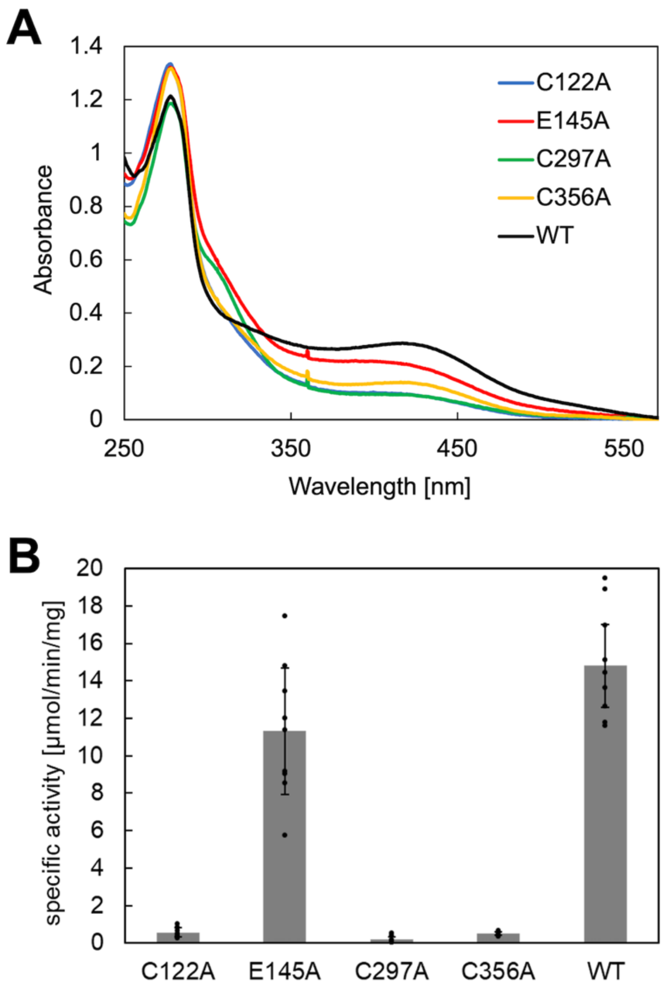


**Supplementary Figure 5.** Mutagenetic studies on *Aeropyrum pernix* phosphomevalonate dehydratase (ApPMDh). (A) UV-visible spectra of ApPMDh mutants. (B) Specific activities of ApPMDh mutants. Assays were performed with enzymes that were affinity-purified and then reconstructed.

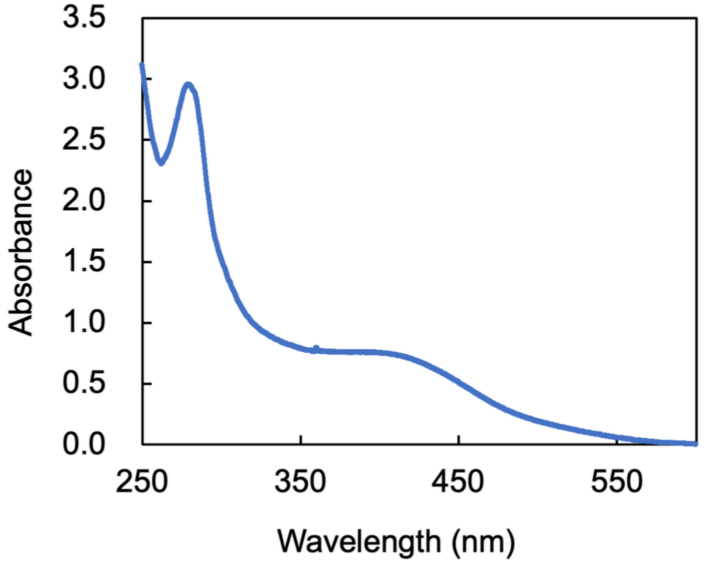


**Supplementary Figure 6.** UV-visible spectra of reconstructed tag-free *Aeropyrum pernix* phosphomevalonate dehydratase (ApPMDh) in glycine-KOH buffer, pH10.
